# An acid-compatible co-polymer for the solubilization of membranes and proteins into lipid bilayer-containing nanoparticles†
†Electronic supplementary information (ESI) available: Experimental procedures; particle size distributions for DLS data and structural parameters used for fitting of SAXS data. See DOI: 10.1039/c8nr01322e


**DOI:** 10.1039/c8nr01322e

**Published:** 2018-05-24

**Authors:** Stephen C. L. Hall, Cecilia Tognoloni, Jack Charlton, Éilís C. Bragginton, Alice J. Rothnie, Pooja Sridhar, Mark Wheatley, Timothy J. Knowles, Thomas Arnold, Karen J. Edler, Tim R. Dafforn

**Affiliations:** a School of Biosciences , University of Birmingham , Edgbaston , Birmingham , B15 2TT , UK . Email: t.r.dafforn@bham.ac.uk; b Diamond Light Source , Harwell Science and Innovation Campus , Didcot , OX11 ODE , UK . Email: stephen.hall@diamond.ac.uk ; Email: tom.arnold@diamond.ac.uk; c Department of Chemistry , University of Bath , Claverton Down , Bath , BA2 7AY , UK; d School of Cellular and Molecular Medicine , University of Bristol , University Walk , Bristol , BS8 1TD , UK; e School of Life & Health Sciences , Aston University , Aston Triangle , Birmingham B4 7ET , UK; f European Spallation Source ERIC , P.O Box 176 , SE-221 00 Lund , Sweden; g Centre of Membrane and Protein and Receptors (COMPARE) , University of Birmingham and University of Nottingham , Midlands , UK

## Abstract

The fundamental importance of membrane proteins in drug discovery has meant that membrane mimetic systems for studying membrane proteins are of increasing interest. One such system has been the amphipathic, negatively charged poly(styrene-*co*-maleic acid) (SMA) polymer to form “SMA Lipid Particles” (SMALPs) which have been widely adopted to solubilize membrane proteins directly from the cell membrane. However, SMALPs are only soluble under basic conditions and precipitate in the presence of divalent cations required for many downstream applications. Here, we show that the positively charged poly(styrene-*co*-maleimide) (SMI) forms similar nanoparticles with comparable efficiency to SMA, whilst remaining functional at acidic pH and compatible with high concentrations of divalent cations. We have performed a detailed characterization of the performance of SMI that enables a direct comparison with similar data published for SMA. We also demonstrate that SMI is capable of extracting proteins directly from the cell membrane and can solubilize functional human G-protein coupled receptors (GPCRs) expressed in cultured HEK 293T cells. “SMILPs” thus provide an alternative membrane solubilization method that successfully overcomes some of the limitations of the SMALP method.

## Abstract 2

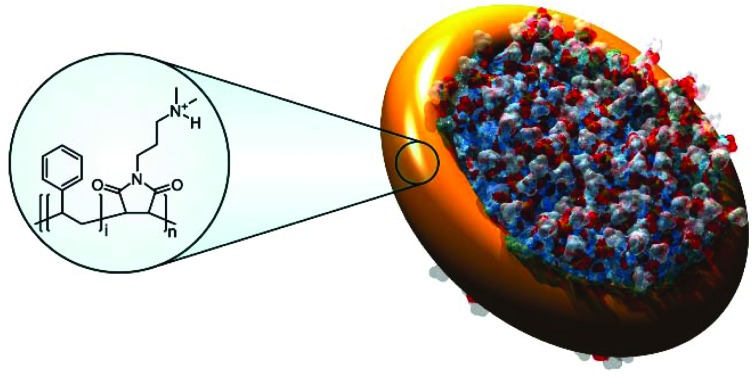
Positively charged poly(styrene-*co*-maleimide) extracts functional membrane proteins into nanodiscs, overcoming some limitations of current nanodisc technology.

## Introduction

With an increasing interest in membrane proteins due to their physiological and pharmacological significance,^1^–^4^ recent developments have yielded alternative solutions to the solubilization bottleneck often limiting purification and characterization.^5^–^10^ A commonly adopted method involves the use of the amphipathic, helical membrane scaffold proteins (MSP),^11^ or peptides inspired by the amino acid residue sequence of the MSP helix,^12^–^14^ to solubilise phospholipid vesicles containing reconstituted membrane proteins into so-called ‘nanodiscs’. These MSP or peptide-stabilized nanodiscs have proven a valuable tool for stabilizing membrane proteins within a planar, nanoscale segment of lipid bilayer surrounded by a proteinaceous belt. MSP nanodiscs have been used extensively for a variety of targets and applications.^10^,^15^,^16^ While it has been observed that peptide-stabilized nanodiscs are potentially more amenable to studying protein complexes within the nanodisc environment,^17^,^18^ they have been shown to have a higher polydispersity than the MSP variety.^14^ However, both these protein-stabilized nanodisc systems still suffer from the limitation that encapsulated membrane proteins need to be extracted using detergent-mediated solubilization before reconstitution into nanodiscs which can lead to instability and disruption of protein–protein interactions. In addition, the peptide nature of the stabilizing belt can lead to spectroscopic interference in downstream applications such as UV circular dichroism (UV-CD) spectroscopy.

**Fig. 1:**
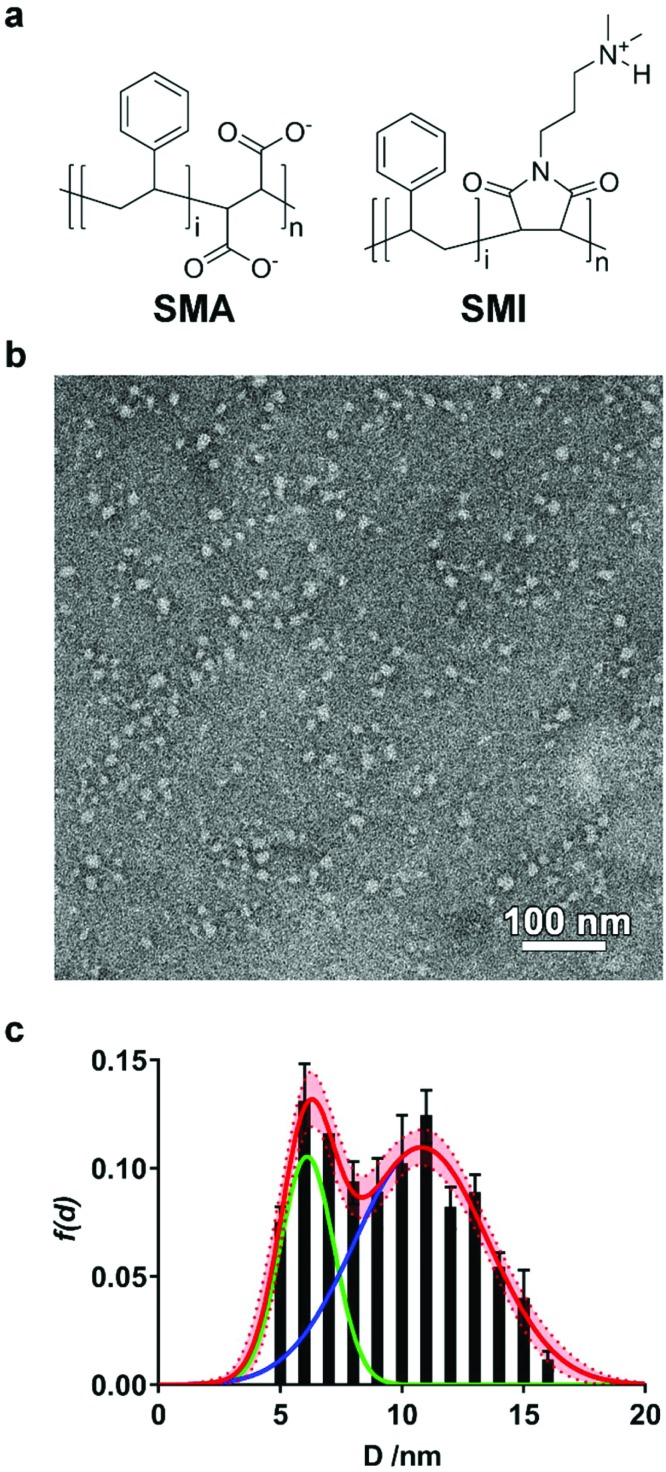
(a) Structures of SMA and SMI. In both cases, *i* = 2*n*. (b) Representative transmission electron micrograph (TEM) of DMPC-SMILPs negatively stained with phosphotungstic acid. (c) Frequency distribution of SMILP diameters imaged with TEM. Bars represent the mean frequency associated with analysis of three separate micrographs with error bars representing ±1 standard deviation. The data fit to the sum of two Gaussian populations of particles (red curve, dashed curve and shaded region represents the standard error associated with nonlinear regression) with maxima at diameters of 6 nm (green line) and 11 nm (blue line).

An alternate strategy is the use of poly(styrene-*co*-maleic acid) (SMA) (Fig. 1a) to extract nanodiscs containing a segment of native cell bilayer, encapsulated by the SMA polymer (termed SMA lipid particles, SMALPs).^19^–^22^ Since the first report of SMA-mediated solubilization^19^ the method has been successfully employed to solubilize a wide variety of targets directly from a range of biological membranes.^23^–^28^ SMALPs have also proven useful in downstream functional^23^,^25^,^29^ and structural characterization.^22^,^24^,^30^–^32^

The commonly used variants of the SMA polymer have now been investigated thoroughly^21^,^33^–^39^ showing that the first used polymer (SMA2000) is the best performing of the polymers so far studied. However, SMA has its limitations. Firstly, the nanodiscs formed by commercially available SMA have a diameter of ∼10 nm 20 which potentially limits the size of proteins that can be solubilized. Secondly, the styrene moiety shows significant absorption of UV light,^40^ which overlaps with the absorption from aromatic residues within proteins. This interferes with spectroscopic techniques to study solubilized proteins. Thirdly, SMALPs have been shown to be unstable in the presence of divalent cations such as Mg^2+^, with precipitation of the polymer occurring under such conditions. Although this can be a useful property under certain circumstances, it can also be a limitation for many potential downstream applications where divalent cations are necessary for membrane protein function.^41^,^42^ Finally, SMA is pH sensitive; at acidic pH values, maleic acid groups become protonated and the polymer becomes insoluble.^39^ This limits the SMALP method to proteins that are stable at basic pHs.

It is both the success and the limitations of the SMA polymer which has spawned a drive to investigate other amphipathic polymers which are capable of solubilizing lipid bilayers into colloidal disc-shaped particles.^43^–^45^ Given the high UV absorbance of styrene, recent advances have been made in establishing the use of styrene-free polymers for nanodisc formation. The first of these copolymers to be investigated was poly(diisobutylene-*co*-maleic acid) (DIBMA) shown to be successful in the solubilization of phospholipid membranes and membrane proteins whilst providing a more native-like phospholipid environment than SMALP nanodiscs.^40^,^46^ Additionally, poly(methacrylate) (PMA) polymers, inspired by the amphiphilic nature of the MSP helix, have been shown to be functional in nanodisc formation.^47^ PMA-stabilised nanodiscs have been applied to stabilization of helical intermediates of amyloid proteins in the presence of a phospholipid membrane. Currently, the efficiency of PMA for solubilization of biological membranes is unknown. In order to address the limited size range of nanodiscs formed by SMA, modification of a low-molecular weight SMA polymer by an amination reaction to form SMA-EA has been shown to form so-called ‘macro nanodiscs’.^48^,^49^ SMA-EA stabilized nanodiscs have been shown to have diameters up to ∼60 nm whilst exhibiting alignment properties in external magnetic fields with demonstrated applications in 2D solid state NMR spectroscopy.

While these new polymers have addressed the issues of UV absorbance, tolerance to low concentrations of divalent cations in solution and size limitations, they are still limited by the same pH constraints as SMA. Two SMA-derived polymers have recently been developed to allow nanodisc formation under acidic conditions. The first such polymer to be developed, SMAd-A, involves modification of SMA with a primary amine. SMAd-A can tolerate low pH and is functional in the solubilization of phospholipids into nanodiscs.^50^ SMAd-A stabilized nanodiscs have been demonstrated to provide an encapsulation platform for the solubilization of hydrophobic drugs in aqueous media. Most recently, modification of SMA with a quaternary ammonium has yielded a polymer (SMA-QA) which is capable of forming nanodiscs.^51^ SMA-QA-stabilized nanodiscs have been shown to remain soluble between pH 2.5 and pH 10, offering a substantial improvement in pH-stability compared to the original SMA polymers. Furthermore, SMA-QA is also able to form macro-nanodiscs of ∼30 nm diameter to overcome the size limitation of SMA-stabilized nanodiscs. However, neither SMAd-A or SMA-QA is commercially available, requiring synthesis through modification of an SMA backbone, and neither have been shown to be compatible with membrane protein solubilization.

Given the limitations discussed above, we have investigated whether an alternative polymer with a similar structure to SMA; poly(styrene-*co*-maleimide) (SMI) (Fig. 1a) can be used in the self-assembly of phospholipid nanodiscs under acidic conditions. SMI is a commercially available amphipathic copolymer of styrene and dimethylaminopropylamine maleimide in a 2 : 1 ratio. SMI has been used to create nanoparticles^52^ capable of oil microencapsulation^53^ and as a surface coating with application to printing.^54^ Despite exploitation of the amphipathic properties of SMI, there have been no reports on SMI-mediated phospholipid solubilization.

Here, we demonstrate that SMI is capable of solubilizing phospholipids by self-assembling in the same way as seen for SMA. We refer to the resulting nanodisc particles as SMI lipid particles (SMILPs). SMI exhibits high thermodynamic efficiency in nanodisc self-assembly which is comparable with SMA. Furthermore, we demonstrate that the size of the lipid core of SMILPs can be tuned as a function of polymer : lipid ratio. SMI circumvents some of the limitations of SMA-mediated solubilization by being tolerant to high concentrations of divalent cations and is soluble in acidic conditions. As SMA is widely used in membrane protein solubilization, we present data to show that SMI is also capable of extracting functional membrane proteins directly from cell membranes without laborious reconstitution following detergent solubilization. These data establish SMI and SMILPs as an alternative to SMA that is an efficient and effective platform for membrane and protein solubilization under acidic conditions.

## Results

### SMI-mediated nanodisc self-assembly

We initially investigated whether SMI in the presence of phospholipids is able to self-assemble into discoidal structures. SMI was added to a suspension of 1,2-dimyristoyl-*sn-glycero*-3-phosphocholine (DMPC) vesicles and analyzed by negative stain transmission electron microscopy (TEM) (Fig. 1b). The absence of vesicles and the clear presence of discrete particles suggests lipid has been solubilized by SMI. A Gaussian analysis of the size distribution in multiple TEM images (Fig. 1c) shows a distribution of diameters with two maxima at 6 ± 1 nm and 11 ± 3 nm. This distribution is similar to that previously determined for SMALP nanodiscs by TEM.^19^,^20^

**Fig. 2:**
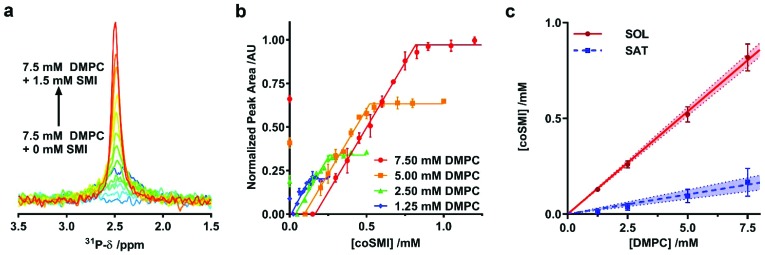
Thermodynamics of SMILP self-assembly. (a) Representative ^31^P-NMR spectra showing an increasing peak area as 7.5 mM DMPC small unilamellar vesicle suspensions are solubilized by increasing SMI concentration from 0 mM SMI (dark blue spectrum) to 1.5 mM SMI (red spectrum). (b) Normalized ^31^P-NMR peak area plotted as a function of polymer concentration with corresponding fits to the experimental data to obtain saturation (SAT) and solubilization (SOL) break points. Each point is the mean of three separate measurements with error bars representing ± standard error. (c) The phase diagram for SMI solubilizing DMPC constructed using SAT and SOL breakpoints determined from b. The SAT boundary is shown as a blue line and the SOL boundary is shown as a red line. Points represent *c*SATs and *c*SOLs breakpoints with error bars representing standard error determined from the fitting procedure in a and b. The shaded region bound by dashed lines represent the 95% confidence bands associated with linear regression.

Having confirmed the capability of SMI to form nanoparticles which conform to the size of previously observed nanodiscs^19^,^20^ we tested the thermodynamic efficiency of SMILP self-assembly using ^31^P-NMR spectroscopy, the principles of which have been described previously^21^,^34^,^37^,^40^ (see Fig. 2). DMPC small unilamellar vesicles (SUVs) alone gave a broad ^31^P peak. Upon addition of SMI at concentrations below that required for the onset of solubilization (the saturation boundary, *c*SATS) this peak broadened beyond detection. This, we believe, is due to low concentrations of SMI causing aggregation of DMPC, rather than solubilization, as previously reported for SMA(3 : 1),^21^,^37^ SMA(2 : 1)^34^ and DIBMA^40^ polymers. Increasing SMI concentration beyond *c*SATS led to the appearance of an isotropic ^31^P peak (Fig. 2a) which linearly increased in area with increasing SMI concentration, corresponding to the proportion of lipids solubilized. Beyond the SMI concentration at which all lipids are solubilized, *c*SOLS, all the lipids are present within a nanodisc phase. Plotting ^31^P peak area against SMI concentration (Fig. 2b) allowed determination of the *c*SATS and *c*SOLS breakpoints at different DMPC concentrations. Plotting the breakpoints obtained as SMI concentration against DMPC concentration (Fig. 2c), enabled definition of the phase diagram for SMI-mediated solubilization of DMPC SUVs. This phase diagram gives the molar ratios of SMI : DMPC required for saturation, *R*b, SATS, and solubilization, *R*m, SATS, from which we can calculate the free energy changes for the vesicle to nanodisc transition associated with DMPC, Δ*G*b→m, 0Lipid, and SMI, Δ*G*b→m, 0Polymer.^21^ These values are compared (Table 1) with equivalent values obtained for two other polymers known to form nanodiscs; SMA2000 and DIBMA. SMA2000 is the SMA polymer variant most similar to SMI structurally and DIBMA is a recently developed polymer.^40^

**Table 1:** Thermodynamic parameters obtained for DMPC-SMILP nanodiscs compared with equivalent data from other nanodisc forming polymers SMA2000^33^ and DIBMA^40^

	SMI	SMA2000	DIBMA
*R* b, SAT S	0.021 ± 0.002	0.050 ± 0.003	0.03 ± 0.005
*R* m, SAT S	0.107 ± 0.013	0.133 ± 0.004	0.062 ± 0.004
Δ*G*b→m, 0Lipid/kJ mol^–1^	0.20 ± 0.07	0.19 ± 0.06	0.077 ± 0.01
Δ*G*b→m, 0Polymer/kJ mol^–1^	–3.90 ± 0.11	–2.23 ± 0.08	–1.76 ± 0.09

### Structural characterisation of SMILP nanodiscs

To this point we have assumed that the structure adopted by SMI-DMPC aggregates is, by analogy to SMA, that of a “nanodisc”. This conclusion is supported by the provisional structural data presented here.

**Fig. 3:**
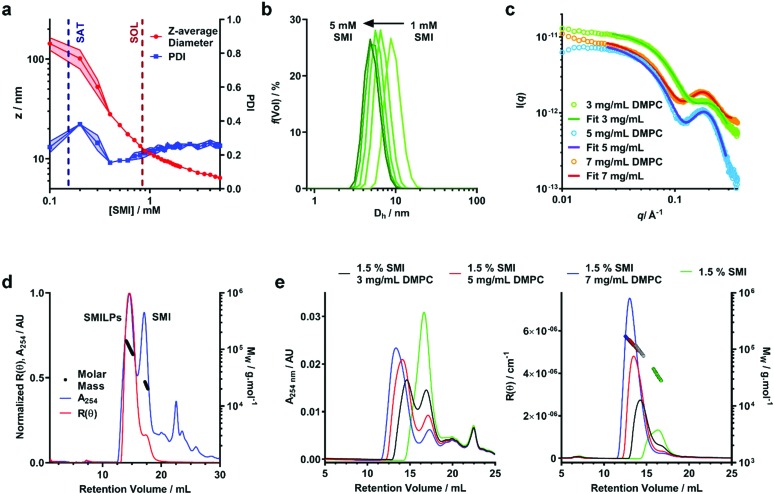
Structural characterization of DMPC-SMILP nanodiscs. (a) DLS data showing the effect of SMI concentration on *Z*-average diameter (red) and polydispersity Index (PDI – blue). SAT and SOL boundaries obtained from ^31^P NMR are shown as dashed blue and red lines respectively. Points represent the mean and shaded regions indicate the standard error obtained from three separate experiments. (b) Volume weighted particle size distribution (PSD) data showing the hydrodynamic diameter (D_h_) for SMILPs formed at SMI concentrations above *c*SOLs. Lines represent the mean PSD of three separate experiments. Error bars are not shown for clarity. (c) Small angle X-ray scattering (SAXS) curves for DMPC-SMILPs made with 1.5% (w/v) SMI with 3, 5 and 7 mg mL^–1^ DMPC (green, blue and red respectively). Points represent the measured scattering intensity, while lines represent the fit to the experimental data. (d) SEC-MALS chromatogram corresponding to SMI-DMPC nanodiscs made with 1.5% (w/v) SMI and 5 mg ml^–1^ DMPC. Traces show the normalized Rayleigh ratio (red trace) and UV absorbance at 254 nm (blue trace) with overlaid mass calculations (black trace). (e) SEC-MALS chromatograms obtained for SMI (green traces) and SMILPs made with 1.5% (w/v) SMI and 3, 5 and 7 mg mL^–1^ (black, red and blue traces, respectively). UV absorbance traces at 254 nm are shown in the left hand graph any Rayleigh ratio traces with overlaid mass calculations (colored circles) are shown in the right hand graph.

We have monitored particle size over the self-assembly process using dynamic light scattering (DLS) (Fig. 3a and S1†). Below the saturation boundary, a large *z*-average diameter was observed. This agrees well with ^31^P-NMR data discussed above, and with previous reports using the SMA(2 : 1),^33^,^34^ SMA(3 : 1)^21^,^37^ and DIBMA^40^ polymers. As polymer concentration increases beyond the SAT boundary, a rapid decrease of *z*-average diameter and polydispersity index (PDI) is observed (Fig. 3a). As mentioned above, we believe this corresponds to the solubilization of the large non-uniform vesicles into SMILPs. Beyond the solubilization boundary, the remaining SMILP aggregates continue to decrease in size by 48% from 11.99 ± 0.26 nm at the SOL boundary to 6.23 ± 0.29 nm diameter at the highest concentration measured. When monitoring the particle size distributions above *c*SOLS (Fig. 3b), a clear shift can be seen towards smaller diameters. These data combined with a relatively constant PDI, suggests that this shift in diameter is not being skewed by excess free polymer in solution. This capacity of SMILPs to be tuneable in size could be beneficial to numerous applications where size is an important parameter.

To improve the structural detail provided by the low resolution DLS data, we have performed small angle X-ray scattering (SAXS) using beamline B21 at Diamond Light Source. We examined SMILPs formed using different polymer : lipid ratios above *c*SOLS (Fig. 3c). The data were fitted with to a polydisperse core–shell bicelle model (see ESI Table S1 and Fig. S2†) which has been used previously to gain structural insight into SMALP nanodiscs,^20^ but with a summed ellipsoid model to account for excess SMI. As can be seen in Fig. 3c, this summed model provided a good fit to the SMILPs. The corresponding fit parameters are shown in Table 2. The structural parameters for DMPC (headgroup and tail sizes and scattering lengths) were fixed based on values from comparable studies.^20^

**Table 2:** Structural parameters obtained through fitting of SAXS data for DMPC-SMILP nanodiscs made with fixed SMI concentration (5.56 mM, 1.5% w/v) and varying DMPC concentrations, in 50 mM sodium acetate, 200 mM NaCl, pH 5. All ratios are above the SOL boundary. Parameters labelled with * were held constant though fitting using values previously determined for DMPC bilayers^55^. The full list of fit parameters is provided in the ESI (Table S1)

DMPC concentration/mg ml^–1^	3	5	7
Molar ratio [SMI] : [DMPC]	1.26	0.75	0.54
DMPC core diameter/nm	1.3 ± 0.2	1.9 ± 0.2	2.3 ± 0.2
SMI belt thickness/nm	1.7 ± 0.2	1.8 ± 0.2	1.6 ± 0.2
DMPC tail length/nm	2.76*	2.76*	2.76*
DMPC headgroup length/nm	0.8*	0.8*	0.8*
Overall diameter/nm	4.7 ± 0.6	5.5 ± 0.6	5.6 ± 0.6

The mass of the SMILP nanodiscs was also investigated by size exclusion chromatography with multiple angle light scattering (SEC-MALS)^56^ (Fig. 3d). These data show negligible aggregated material since there is no strong signal eluting from the column at the 8 mL void volume. After this, two major peaks eluted, the first of which shows both strong light scattering intensity, indicating the presence of large particles, and UV absorption signals, indicating the presence of styrene from SMI. We have assigned this peak to SMILPs. It gives a mass averaged *M*_w_ of 104.7 ± 0.8 kDa, a number averaged *M*_n_ of 102.8 ± 4.4 kDa, resulting in a PDI of 1.02. In-line DLS further confirms the presence of SMILPs; giving a hydrodynamic diameter of 8.68 ± 0.87 nm, consistent with the range of SMILP diameters observed by independent DLS. The second peak shows a strong UV absorbance yet low scattering intensity. The *M*_w_ and *M*_n_ of this peak are 26.7 ± 3.5 kDa and 26.3 ± 3.31 kDa respectively, giving a PDI of 1.01. A hydrodynamic radius was measured to be 7.44 nm. This peak has been assigned to excess SMI polymer aggregates in solution by account of the decreased scattering intensity yet strong UV absorption. Further downstream peaks were also seen with UV yet gave no discernable scattering intensity. We propose these peaks are due to the presence of short oligomeric polymers which are present as a by-product of SMI synthesis.

To provide further evidence of the size-tuneability of SMILPs, SEC-MALS was performed on 1.5% SMI and SMILPs made at the same SMI : DMPC ratios as measured by SAXS (Fig. 3e). SEC-MALS of SMI in the absence phospholipids confirms our earlier assignment of the second peak in Fig. 3d being due to SMI aggregates. SMILPs formed at higher SMI : DMPC ratios show the same trend as observed by SAXS and DLS, exhibiting higher retention volumes on the column, indicating formation of smaller particles. The *M*_W_ calculations from light scattering data and hydrodynamic diameter measured by in-line DLS also agree with this assertion, with SMILPs formed at a higher SMI : DMPC ratio having a both a lower mass and smaller diameter (Table S2†). In addition, the ratio of the UV peaks corresponding to SMILPs and SMI decreases indicating that at higher DMPC concentrations, more SMI associates with the nanodiscs while the proportion of SMI forming lipid-free aggregates in solution decreases.

### Stability of SMILPs

**Fig. 4:**
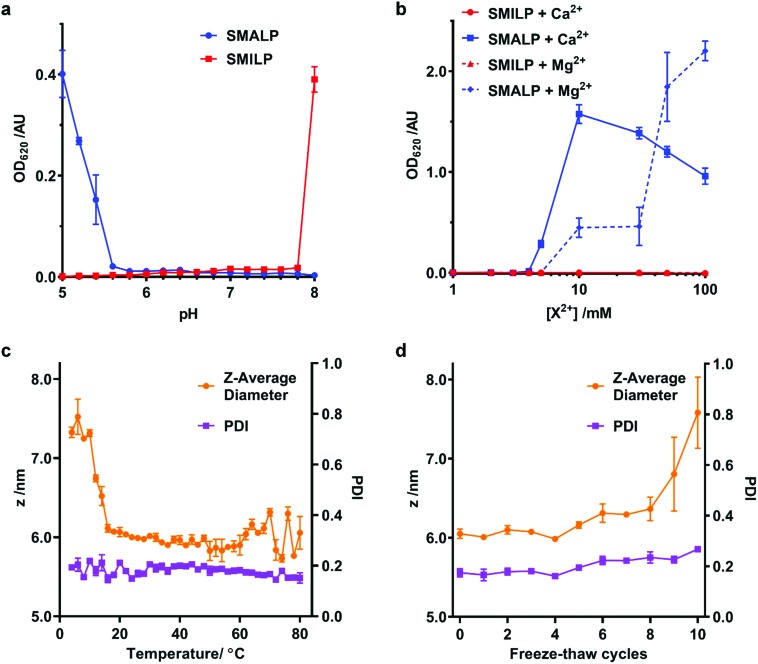
Stability of SMILPs. (a) The turbidity of SMALP and SMILP solutions as a function of pH. Data was recorded at concentrations of 0.33 mg mL^–1^ DMPC, 0.1% w/v SMA or SMI for SMALPs and SMILPs respectively, in 50 mM sodium phosphate, 200 mM NaCl. (b) The turbidity of SMALP and SMILP solutions in response to increasing concentrations of Mg^2+^ or Ca^2+^. (c) DLS data showing the effect of temperature on *Z*-average diameter (magenta) and PDI (green) of DMPC-SMILPs (1.5% w/v SMI, 5 mg mL^–1^ DMPC, 50 mM sodium acetate, 200 mM NaCl, pH 5). Points represent the mean value taken from three separate experiments with error bars displaying ±1 standard error. (d) DLS data showing the effect of freeze–thaw cycles on *Z*-average diameter (magenta) and PDI (green) of DMPC-SMILPs (1.5% w/v SMI, 5 mg mL^–1^ DMPC, 50 mM sodium acetate, 200 mM NaCl, pH 5). Points represent the mean value taken from three separate experiments with error bars displaying ±1 standard error.

A limitation of SMA in many downstream biological applications is the low tolerance to divalent cations and insolubility at low pH. The polyimide structure of SMI inherently means that it behaves differently to SMA. We therefore directly compared the stability of SMALPs and SMILPs in the presence of Ca^2+^ or Mg^2+^ or as a function of pH (Fig. 4). This was done by measuring the turbidity of nanodisc solutions (Fig. 4a & b). The data show that SMALPs begin to precipitate at 5 mM Mg^2+^ and 4 mM Ca^2+^, as indicated by an increased turbidity, with severe precipitation occurring above 10 mM in both cases. SMILPs, however, can tolerate concentrations of Mg^2+^ and Ca^2+^ in excess of 100 mM. The pH dependence for the two polymers shows an intuitive trend; SMALPs are only soluble at pH values >5.8, but SMILPs are only soluble below pH 7.8. This gives SMILPs a broad working pH range, and importantly allows studies at physiological pH (7.4).

In addition, SMILPs exhibit high thermal stability as monitored by DLS (Fig. 4c & d). We observed no significant changes to polydispersity up to 80 °C and no changes in diameter over a wide temperature range (16 °C–80 °C) However, there was a decrease in diameter between 10 °C and 16 °C. Importantly, we observed no signs of aggregation or precipitation at elevated temperature. We also observed that the diameter of the particles increased from 6.05 ± 0.10 nm to 7.58 ± 0.78 nm after multiple freeze–thaw cycles. This is not enough to suggest aggregation or precipitation and no peaks of larger diameter were observed in the associated particle size distributions (see ESI, Fig. S3†). These data indicate that SMILPs are a stable platform which is ideal for applications involving the presence of divalent cations and acidic pH.

### SMILP solubilization of membrane proteins from biological membranes

**Fig. 5:**
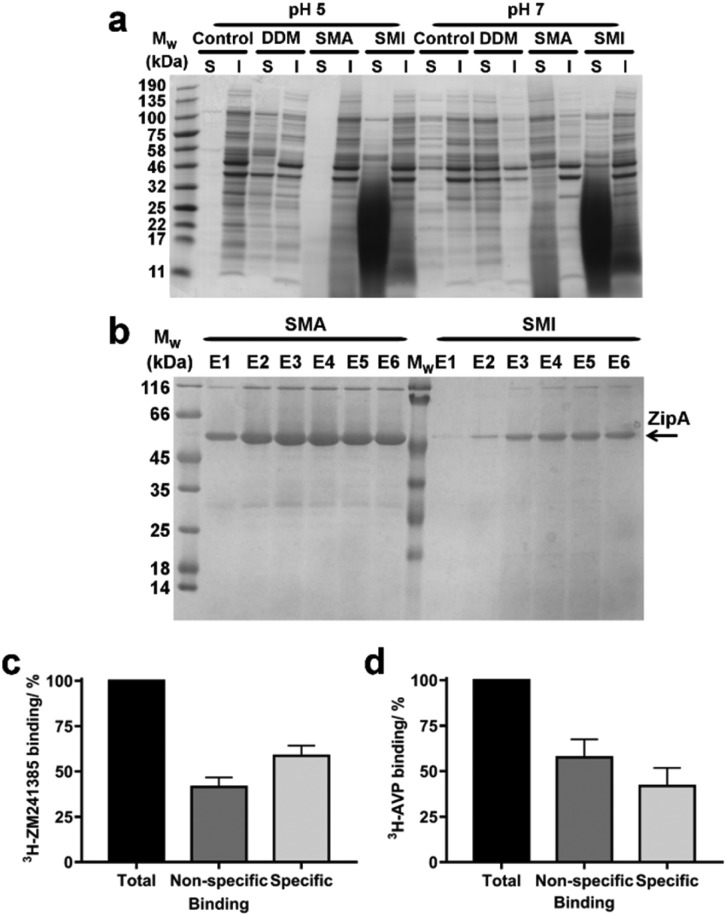
The solubilization of membrane proteins using SMI. (a) Coomassie stained SDS-PAGE showing the range of proteins that have been solubilized (S) and remained insoluble (I) after the incubation of *E. coli* membranes with SMI compared to DDM SMA2000 at pH 5 and pH 7. A control using membranes without the addition of SMI, DDM or SMA is also shown. (b) Coomassie stained SDS-PAGE showing the relative yield and purity of *E. coli* His_6_-ZipA extracted with SMA compared with SMI over six elutions (E1–E6) from Ni^2+^-NTA IMAC purification. (c) Binding of [^3^H]ZM241385 to A_2A_R-SMILP extracted from HEK 293T cells expressing human A_2A_R. Non-specific binding was defined by a saturating concentration (1 μM) of ZM241385. (d) Binding of [^3^H]vasopressin ([^3^H]AVP) to V_1a_R-SMILP extracted from HEK 293T cells expressing human V_1a_R. Non-specific binding was defined by a saturating concentration (1 μM) of AVP. Binding data are mean ± s.e.m. of three separate experiments performed in triplicate with total binding, non-specific binding and specific binding shown in each case.

SMA has been widely utilized for the solubilization of membrane proteins. We therefore investigated the efficiency of SMI to solubilize membrane proteins directly from native *Escherichia coli* (*E. coli*) membranes. The solubilizing capability of SMI was compared to that of SMA2000 and to the detergent n-dodecyl β-d-maltoside (DDM), which is commonly employed for solubilizing functional membrane proteins. A control where no solubilizing agent was added to the membranes was also included (Fig. 5a). Soluble and insoluble fractions were separated by ultracentrifugation and analyzed by SDS-PAGE. SMILP-solubilized samples show a diffuse low molecular weight band that results from the presence of polymer in the soluble fractions at pH 5 and pH 7. Despite this, it is clear that both SMI and DDM effectively solubilize *E. coli* membrane proteins, which are evident in the supernatant fraction (S) at both pH values (Fig. 5a). In contrast, SMA is ineffective at pH 5. At pH 7 however, both SMI and SMA effectively solubilized membrane proteins, consistent with the pH-dependence data of SMILP and SMALP stability presented in Fig. 4a. However at pH 7, SMI is less effective than SMA or DDM.

In order to be widely adopted, SMILP-solubilized proteins must be amenable to purification techniques. To investigate the yield and purity of SMILP-solubilized compared to SMALP-solubilized proteins, Ni^2+^-NTA immobilized metal affinity chromatography (IMAC) was performed on *E. coli* ZipA, which has been previously used to determine the efficiency of different SMA polymers,^38^ after extraction with SMA and SMI (Fig. 5b). The yield obtained when extracting ZipA using SMI is marginally less than when using SMA, which is corroborated by whole membrane extractions discussed above. Importantly, SMILP-ZipA can be obtained at a higher purity than SMALP-ZipA.

To investigate if the functional capability of membrane proteins was preserved following encapsulation in a SMILP, two G-protein-coupled receptors (GPCRs) were SMILP-solubilized. The human adenosine A_2A_ receptor (A_2A_R) and the human V_1a_ vasopressin receptor (V_1a_R) were each transiently expressed in HEK 293T cells and the cells solubilized by SMI. The binding capability of the SMI-solubilized membranes containing GPCR-SMILPs was determined by radio-ligand binding assays using [^3^H]ZM241385 as tracer ligand for the A_2A_R encapsulated in a SMILP (A_2A_R-SMILP) and [^3^H]vasopressin ([^3^H]AVP) as tracer ligand for the V_1a_R-SMILP. Non-specific binding was determined in each case by a saturating concentration (1 μM) of unlabeled ligand. The SMILP-solubilized A_2A_R and V_1a_R were both functional as specific binding to the receptor was observed for both the A_2A_R-SMILP (Fig. 5c) and the V_1a_R-SMILP (Fig. 5d), with 59 ± 6% and 42 ± 10% (mean ± s.e.m.) of total binding being specific binding, respectively.

## Summary and discussion

The similarity in the thermodynamic parameters for SMALP self-assembly to those obtained for SMI show that there is a similar thermodynamic driving force for the formation of both SMALPs and SMILPs. In both cases it is the polymer that drives this process, despite the electrostatic differences between SMI and SMA. SMI shows a slightly larger negative free energy change upon interaction with the lipids than SMA, indicating a more favorable self-assembly. As with all other polymers so far studied (SMA(2 : 1),^33^,^34^ SMA(3 : 1)^21^,^37^ and DIBMA^40^) there is a small positive free energy change associated with DMPC moving from vesicle to nanodisc. This means that SMI can be thought of as a mild membrane solubilizer, since it effectively keeps the lipid molecules in a free-energy environment similar to that experienced in vesicles.

When comparing SMI to DIBMA, SMI is more efficient at initiating the onset of solubilization, although higher concentrations of SMI are required for completion of solubilization than that of DIBMA. The free energy change associated with DMPC during nanodisc formation is slightly less positive in “DIBMALPs” than in SMILPs. The driving force for SMI to self-assemble into nanodiscs is over twice as strong as DIBMA, indicating SMILP formation is overall more thermodynamically favored in comparison to DIBMALPs.

SMALPs have been widely reported to have diameters of approximately 10 nm. Data presented here suggest that SMILPs appear slightly smaller. By comparison, pH-resistant SMA-QA polymers form ‘macro-nanodiscs’ with substantially larger diameters up to approximately 30 nm (ref. 51) while the acid-soluble SMAd-A has been observed to form nanodiscs slightly smaller than observed for SMALPs: between 5–10 nm.^50^ Values from TEM, DLS and SEC-MALS suggest SMILPs have diameters ranging from 12 to 6 nm, similar to SMAd-A nanodiscs. A more in depth structural investigation of SMILPs using SAXS suggest a smaller overall particle diameter of around 5 nm. This discrepancy in SMILPs analyzed by TEM appearing larger is likely due to negative staining by PTA coating the particles, leading to a larger apparent diameter. DLS and SEC-MALS are similarly affected by the hydration shell around SMILPs, with analysis assuming a spherical particle. SAXS is insensitive to these limitations, providing a more accurate estimation of size, whilst also showing that the adopted structure is consistent with the ‘nanodisc’ model proposed for SMALPs. The presence of free SMI as determined by SAXS and SEC-MALS may also explain the particle size distribution seen by TEM. The population of smaller particles is most likely due to aggregates formed by excess SMI. This is similar to previous studies of oil microencapsulation by SMI. However, it is also possible that the second population also represents the second dimension of the SMILP nanodisc structure (*i.e.* it is slightly bigger than the “thickness” of the nanodiscs determined by SAXS: lipid tail + lipid head is ∼3.5 nm).

The parameters obtained from fitting SAXS data, taking into account the presence of free SMI, indicate that SMILPs are smaller than SMALPs due to a decreased diameter of the phospholipid core. The diameter of the core, however, increases slightly with an increased DMPC : SMI ratio while the thickness of the SMI belt remains unchanged. These data are in agreement with DLS data, suggesting that phospholipid partitions between the available polymer, leading to changes in particle diameter for different relative ratios of DMPC : SMI. Interestingly, SEC-MALS data collected for SMILPs formed at different SMI : DMPC ratios indicate that once formed, excess SMI aggregates can be removed and the different sizes of SMILPs can be maintained through purification. This could have important application to membrane protein solubilization whereby the size of the SMILP can be tuned to optimally accommodate the target protein.

Two of the major limitations of SMALPs have been their insolubility at acidic pH and their very low tolerance of divalent cations. SMILPs display the opposite pH dependence to SMALPs, whilst being able to tolerate higher concentrations of divalent cations than SMALPs and DIBMALPs. Meanwhile, SMILPs show improved thermal stability relative to SMALPs,^33^ with demonstrated stability up to 80 °C and over many freeze–thaw cycles. While a decrease in diameter is observed between 10 and 16 °C, the mechanism behind this change remains unclear. While it could be indicative of minor lipid loss from increased thermal motion of either the lipids or the polymer, it is also possible that more subtle structural rearrangements occur to which the DLS technique is insensitive. However, regardless of the mechanism of this small change in SMILP size, the absence of any large aggregates and relatively constant PDI up to 80 °C indicate SMILPs provide a stable platform for membrane protein solubilization.

When used to solubilize membrane proteins from biological membranes, SMI is effective below pH 7.8 and importantly is effective at the physiological relevant pH of 7.4. This is an important attribute as it allows membrane proteins that cannot tolerate low pH to be studied using SMILPs. The decreased solubilization efficiency of SMI compared to SMA at pH 7 may be due to the size of SMILPs. With such a small lipid core, it is unclear how much native lipid can remain in the SMILP in addition to a membrane protein. SMI does not exhibit any selectivity towards the size of the proteins which are solubilized within SMILPs, with both high and low *M*_w_ proteins being equally represented. This is surprising given the smaller diameter of the lipid core as determined by SAXS. Although SMI forms nanodisc structures in the presence of phospholipids alone, the addition of membrane proteins may lead to SMI acting more as an amphipol rather than a nanodisc, but with the benefit of being able to extract proteins directly from cell membranes. Irrespective of the solubilization mechanism, it is clear that the SMILP-solubilized GPCRs investigated here retain a conformation capable of ligand binding. The A_2A_R and the V_1a_R have different binding modes in that the A_2A_R binds small biogenic amine ligands within the transmembrane helical bundle whereas the V_1a_R binds larger nonapeptides to a binding site comprising extracellular elements in addition to the helical bundle.^57^ Despite differences in their binding modes, both A_2A_R-SMILP and V_1a_R-SMILP retained ligand binding capability. In each case non-specific binding of radio-ligand was expected, resulting from low level ligand partitioning into the lipid bilayer, or the SMI polymer belt. However, the degree of non-specific binding when using GPCR-SMILPs is similar to previously observed for GPCR-SMALPs and low enough to allow accurate measurement of specific binding. The non-specific binding of [^3^H]ZM241385 to A_2A_R-SMILP was 41 ± 5.3% of total binding, compared to 39 ± 3.8% observed for [^3^H]ZM241385 binding to the same receptor in a SMALP (A_2A_R-SMALP).^23^ Consequently, using GPCRs as an example, it has been shown that SMI-solubilized membrane proteins remain functional. Furthermore, SMILP-solubilized ZipA can be obtained to a higher degree of purity than SMALP-solubilized ZipA, albeit at a slightly lower yield. This could have important implications for applications such as electron microscopy, where only micrograms of sample are required but at a very high level of purity.

## Conclusions

Nanodisc technology is becoming widely adopted as a membrane and membrane protein solubilization strategy. The recent development of SMALP nanodiscs provides benefits over other alternative solubilization strategies by being able to solubilize membrane proteins directly from the host cell membrane whilst keeping the annular lipids present within the nanodisc to maintain the native environment of the membrane protein.^29^ SMALPs however are limited by two predominant factors: insolubility at low pH and precipitation in the presence of divalent cations.

We have presented data showing that the positively charged SMI polymer can self-assemble in the presence of phospholipids in acidic conditions to form SMI lipid particle (SMILP) nanodiscs which are both thermally stable and stable in the presence of divalent cations. SMI is also capable of extracting functional membrane proteins directly from biological membranes.

Recent developments in nanodisc-forming polymers have resulted in a range of polymers capable of forming nanodiscs which can solubilize membrane proteins. These polymers, now with the addition of SMI, provide a nanodisc toolbox for the study of membrane proteins, where the nanodisc can be tuned to application. For example, SMA has been successfully utilised for structural studies of membrane proteins by electron microscopy,^24^,^32^ X-ray crystallography,^31^ and solid-state NMR^30^ as well as functional studies.^23^,^25^,^29^ However, the limitations discussed above still apply. For solubilization of larger membrane proteins or complexes, a larger nanodisc may be required. High resolution cryo-transmission electron microscopy also benefits from a larger particle. It has been demonstrated that SMA polymers synthesised by random addition fragmentation chain transfer (RAFT) polymerisation can form larger nanodiscs^43^ which would be suited for these studies. Similarly, macro-nanodiscs formed by SMA-QA^51^ and SMA-EA^48^,^49^ may prove advantageous for study of membrane proteins by electron microscopy. In addition, structural studies of membrane proteins by 2D solid-state NMR could benefit from the demonstrated magnetic alignment properties of SMA-QA and SMA-EA nanodiscs. For downstream spectroscopic applications such as circular dichroism, DIBMA^40^ and PMA^47^ are ideal polymers due to the absence of the styrene moiety leading to non-overlapping UV-absorbance signals from the polymer and solubilized proteins. Sulfhydryl-modified SMA (SMA-SH)^44^ is capable of solubilizing membrane proteins and is ideal for fluorescence studies and surface-coupling applications such as surface plasmon resonance, where specific chemistries can be easily added to the polymer. Now, we have shown that SMI is the ideal polymer for the solubilization of membrane proteins that require acidic pH or the presence of high concentrations of divalent cations directly from biological membranes. For example, membrane-associated ATPase enzymes^41^ and the calcium dependant potassium channel superfamily^42^ require Mg^2+^ or Ca^2+^ for activity, which would be incompatible with the SMALP system.

Having demonstrated the potential of SMILPs, we will continue our studies of this system to obtain more detail on the structure, function and the mechanism by which it operates. As a first step we have already performed neutron small angle scattering measurements (data currently under analysis) that will provide improved structural detail on the size and shape of SMILPs.

Taken together these data show that SMILPs address some of the long-standing limitations of SMALPs and other existing nanodisc forming polymers. Together this makes SMILPs and important addition to the membrane nanodisc toolbox.

## Conflicts of interest

There are no conflicts to declare.

## Supplementary Material

Supplementary information
