# Supplementary material for: An acid-compatible co-polymer for the solubilization of membranes and proteins into lipid bilayer-containing nanoparticles
Source: Nanoscale. 2018 May 24;10(22):10609–19. doi: 10.1039/c8nr01322e (PMC5996351; doi:10.1039/c8nr01322e)
Supplement: Supplementary file 1 [file NR-010-C8NR01322E-s001.pdf]

Electronic Supplementary Material (ESI) for Nanoscale.
This journal is © The Royal Society of Chemistry 2018

Electronic Supplementary Information

An Acid-Compatible Co-Polymer For the Solubilization of Membranes and Proteins Into Lipid Bilayer-containing Nanoparticles

Stephen C. L. Hall[*a,b], Cecilia Tognoloni[c], Jack Charlton[a], Éilís C. Bragginton[d], Alice J. Rothnie[e], Pooja Sridhar[a], Mark Wheatley[a], Timothy J. Knowles[a], Thomas Arnold[§,b,c,f], Karen J. Edler[c] and Tim R. Dafforn[§,a]*

[a] School of Biosciences, University of Birmingham, Edgbaston, Birmingham, B15 2TT, UK

[b] Diamond Light Source, Harwell Science and Innovation Campus, Didcot, OX11 ODE, UK

[c] Department of Chemistry, University of Bath, Claverton Down, Bath, BA2 7AY, UK

[d] School of Cellular and Molecular Medicine, University of Bristol, University Walk, Bristol, BS8 1TD, UK

[e] School of Life & Health Sciences, Aston University, Aston Triangle, Birmingham B4 7ET, U.K

[f] European Spallation Source ERIC, P.O Box 176, SE-221 00 Lund, Sweden

*** Corresponding author. Email: stephen.hall@diamond.ac.uk**

[§.] Alternative contacts: t.r.dafforn@bham.ac.uk; tom.arnold@diamond.ac.uk

MATERIALS AND METHODS

Materials

SMI was purchased as SMA2000I resin from Cray Valley (UK). SMA2000I has a weight averaged molecular weight, M_w of 7500 and a number averaged molecular weight, M_n of 2700. SMA was purchased as SMA2000 (poly(styrene-*co*-maleic anhydride) SMAnh) from Cray Valley (UK) with an M_w of 7500 and an M_n of 3000. 1,2-dimyristoyl-*sn*-glycero-3-phosphocholine (DMPC) was purchased as a powder from Avanti Polar Lipids (USA). 400 mesh carbon coated Cu grids were purchased from Agar Scientific (UK). Precast Criterion XT graduated 4 – 12 % bis-tris poly(acrylamide) gels were purchased from Bio-Rad Laboratories Ltd. (UK). InstantBlue protein stain was purchased from Expedion (UK). [^{3}H]ZM241385 (specific activity 50 Ci/mmol) was purchased from American Radiolabelled Chemicals (Cardiff, UK). [Phe-3,4,5-^{3}H]AVP (specific activity 68 Ci/mmol) was supplied by PerkinElmer (Stevenage, UK). ZM241385 (4-(2-[7-amino-2-(2-furyl)[1,2,4]triazolo[2,3-a][1,3,5]triazin-5-yl amino]ethyl)phenol) was purchased from Tocris (Bristol, UK) and AVP was from Bachem (Weil am Rhine, Germany). The standard HEK 293T cell line was provided as a kind gift from Professor John Heath (University of Birmingham, UK). All other chemicals and reagents were purchased from Sigma-Aldrich (UK) at > 98 % purity and used without further purification.

Methods

Solubilization of SMI and SMAnh

SMA2000I resin was solubilized by following the manufacturer's instructions. In brief, concentrated HCl was added dropwise to a 10 % suspension of SMA2000I in ultrapure water to a concentration of 1 M. This solution was heated under reflux at 125 ℃ for 2 – 4 hours until the solution has clarified. Solubilized SMI was then precipitated by the addition of 5 M NaOH to pH 9.0 and washed three times in ultrapure water. Precipitated SMI was dissolved in a minimal volume of 0.6 M HCl, the pH adjusted to as required and lyophilized. SMI stocks were made directly from the dried powder.

SMAnh (poly(styrene-*co*-maleic anhydride)) was hydrolyzed from SMA2000 resin as previously described [1]. Briefly, a 10 % w/v suspension of SMA2000 resin in 1 M sodium hydroxide

was heated under reflux at 125 °C for 2 hours until the solution clarified. Solubilized copolymer was precipitated after cooling by lowering the pH to 5.0 with the drop-wise addition of concentrated hydrochloric acid and then washed 3 times in ultra-pure water. Washed, precipitated SMA was re-dissolved in 0.6 M NaOH overnight and the precipitation and washing procedure repeated. Finally, SMA was dissolved in a minimal volume of 0.6 M NaOH, pH adjusted to 8.0 and lyophilized. SMA stocks were made directly from the dried powder.

Nanodisc Preparation

DMPC was weighed and dissolved in chloroform in glass vials. A lipid film was deposited on the surface of the vials by evaporating chloroform with N_2 and then desiccating overnight to remove trace solvent. DMPC was hydrated to 10 mg/mL with buffer (50 mM NaOAc, 200 mM NaCl, pH 5 for SMILPs and 50 mM sodium phosphate, 200 mM NaCl, pH 8 for SMALPs) at 25 °C and sonicated in a water bath for 30 minutes until the suspensions turned clear, indicating the formation of small unilamellar vesicles (SUVs). DMPC SUV suspensions were stored at room temperature and used within 3 days.

SMI stock solutions were made in 50 mM sodium acetate, 200 mM NaCl, pH 5 to the required molar concentration (assuming no changes in molecular weight distribution upon copolymer solubilization) based on the M_n value for titration experiments. For experiments using nanodiscs made at excess copolymer concentration, SMI stock solutions were made to 3 % w/v in the same buffer. SMA stock solutions were made to 3 % w/v in 50 mM sodium phosphate, 200 mM NaCl pH 8.

DMPC nanodiscs were made by mixing DMPC and copolymer stock solutions in a 1:1 ratio giving final concentrations of 5 mg/mL DMPC, 1.5 % w/v SMI or SMA. For titration experiments, stock solutions were diluted and mixed as required to give the correct final concentrations. Nanodiscs were left at 25 °C for at least 16 hours to ensure equilibration prior to use.

Negative Stain Transmission Electron Microscopy (TEM)

400 mesh carbon coated Cu grids were glow discharged twice for 15 seconds with a 15 second pause between charges. SMILP solutions were diluted to 0.1 mg/mL DMPC, 0.03 % w/v SMI in 50 mM NaOAc, 200 mM NaCl, pH 5 and centrifuged at 16,000 × *g* for 10 minutes to remove particulate contaminants. 5 µL of diluted SMILP solutions were added to the glow discharged grids and allowed to adsorb for 1 minute. The grids were washed three timed with ultra-pure water

and stained twice successively for 1 minute with 1 % w/v phosphotungstic acid. Excess liquid was removed from the grids at each stage by blotting with filter paper.

Samples were imaged on a Tecnai T20 twin-lens transmission electron microscope (FEI, Cambridgeshire, UK) operating at an accelerating voltage of 200 kV. Images were recorded at a magnification of × 62,000 at -1.5 µm under focus. Images were recorded on an Eagle 4k × 4k CCD camera (FEI, Cambridgeshire, UK).

Subsequent image analyses were carried out in ImageJ (FIJI). A total of 1038 particles were analyzed from micrographs where staining was of high quality to allow for reliable particle picking.

^{31}P NMR Spectroscopy

^{31}P NMR was carried out essentially as previously described,[2] but with the following modifications. SMI and DMPC mixtures were prepared as described above in 50 mM sodium acetate, 200 mM NaCl, pD 5.0 using DMPC concentrations of 7.50, 5.00, 2.50 and 1.25 mM. The M_n value for SMA2000I was used to calculate molar concentrations of SMI, as the distribution was assumed to remain unchanged throughout the solubilization procedure described above. ^{31}P NMR spectra were recorded at 298 K using an Avance III 400 MHz NMR spectrometer (Bruker, UK) operating with an excitation frequency of 161.98 MHz using ^{1}H-decoupling. 256 scans were recorded per measurement over a sweep width of 32051 Hz, with an acquisition time of 1.022 s, a pre scan delay of 6.5 s, a relaxation delay of 5 s and a pulse width of 7.25 µs. Spectra were referenced to an external standard of 85 % H_3PO_4 in 10 % D_2O to correct for any changes in field strength between measurements.

Thermodynamic Calculations

The thermodynamic analysis performed is based on the work of Vargas *et al.*[3], and has been described by them and us[2] previously. Briefly, to obtain saturation and soluilization breakpoints (c_S^{SAT} and c_S^{SOL}, respectively) the ^{31}P-NMR peaks were integrated using TopSpin software (Bruker, UK) and the absolute integrals normalized to the largest and smallest value in each data set. Normalized integrals were then averaged from three independent measurements, plotted as a function of SMI molar concentration and fit to the scenario described previously[2–6]. This fitting procedure yields c_s^{SAT} and c_S^{SOL} breakpoints, ie, the concentration of SMI required for the onset and completion of solubilization at each DMPC concentration. Plotting these breakpoints as molar

concentrations of SMI against DMPC yields the phase boundary lines. The gradient of each of these lines describes the molar ratio of SMI to DMPC required for the vesicle saturation, $R_{S}^{b,SAT}$, and solubilization, $R_{S}^{m,SOL}$. These molar ratios allow the calculation of partitioning coefficients for SMI and DMPC which in turn allow for the calculation of the free-energy of transition of both DMPC, $\Delta G_{Lipid}^{b \rightarrow m,0}$, and SMI, $\Delta G_{Polymer}^{b \rightarrow m,0}$, transitioning from a vesicle/aggregate to a nanodisc. A detailed description of this analysis has been published previously[3].

Dynamic Light Scattering

Dynamic light scattering experiments were performed as previously described [2]. In brief, samples were loaded into 45 μL quartz cuvettes with a 3 × 3 mm light path (Hellma Analytics, Germany). Measurements were taken using a Zetasizer Nano S (Malvern Instruments, Worcestershire, UK) equipped with a He-Ne laser at 633 nm with a detector angle of 178° relative to the incident beam. All measurements were performed after equilibrating the sample at 25 °C for 60 seconds. Each sample measured 3 times with the attenuator position automatically optimized for size determination. Each measurement consists of 11 scans of 10 seconds. Freeze-thaw stability was performed by flash freezing the nanodisc solution in liquid N_2 for 5 min and then thawing at room temperature before taking the sample to load into the cuvette. Temperature stability was performed by increasing the temperature from 4 to 80 °C in 1°C increments. The samples were measured at each temperature after equilibrating at that temperature for 2 minutes. Data analysis was performed after taking into account the viscosity and refractive index of all buffer constituents by fitting a non-negatively constrained least squares function to the measured autocorrelation function. This gives an intensity weighted particle distribution assuming spherical particles, which is converted to a volume weighted particle size distribution using Mie scattering theory[7,8]**.** A volume weighted PSD takes into account the increased scattering of light by larger particles to give a more realistic representation of the particles present. Cumulant analysis was also performed to obtain the Z-average diameter and polydispersity index[9]**.** The polydispersity index (PDI) is defined as the square of the ratio of the peak value to the width of the Gaussian distribution obtained from cumulant analysis.

Small Angle X-ray Scattering (SAXS)

SMILP samples were prepared as described above, at a final solution concentration of 1.5wt% SMI, with 3, 5 or 7 mg/ml DMPC in a pH 5 acetate buffer containing 0.2 M NaCl. Samples were

not gel filtered prior to measurement. SMILP solutions, a solution of the SMI polymer alone at 1.5 % (w/v) in the same buffer and the buffer were placed in a 96-well plate at 25 °C, and loaded into the Arinax BioSAXS robot sample changer on the SAXS beamline B21 at Diamond Light Source. Solutions were measured using the standard beamline configuration, at 12.4 keV, in a 1mm diameter quartz capillary, that was automatically washed, dried, and flushed with buffer before each measurement. Measurements were taken as 60 frames of 1 second using a Pilatus 2K detector. The buffer solution was measured before and after each sample solution in the same capillary, and background scattering was subtracted from the data. Data was measured over a q range of 0.008 to 0.4 $Å^{-1}$, calibrated using silver behenate and reduced using the data reduction pipeline in DAWN[10].

Fitting of SAXS Data

The data was fitted in the NIST SANS Analysis package (reference: Kline, S. R., Reduction and Analysis of SANS and USANS Data using Igor Pro. J Appl. Cryst. 2006, 39 (6), 895.) within Igor Pro (Wavemetrics) to a summed model consisting of one component to account for scattering from the SMILPs, and one from free polymer in solution. The solutions were not gel filtered prior to measurement but subsequent gel filtration measurements indicated the presence of free polymer. Therefore, scattering from the polymer in solution, which had been measured separately under the same buffer, salt and polymer concentrations (0.2 M NaCl, 50 mM acetate buffer, 1.5 % (w/v) polymer) was added to the scattering from the SMILPs. The dimensions of the polymer aggregates in polymer-only solutions in the same buffer were initially fitted to an ellipse model[11]. The values obtained for the radii of the polymer-only aggregates were then initially held during fitting of the scattering from the SMILP solutions. The scale factor (proportional to the relative concentration of these polymer-only objects) was allowed to vary. The SMILP component of the scattering was fitted to the polydisperse core-shell bicelle model[12] previously used for fitting scattering data from SMALPs[13]. This model, shown in Figure S3, contains a central cylindrical lipid bilayer region (core) modeled as an inner cylinder region composed of the lipid tails, with a layer at each end corresponding to the hydrated lipid headgroups. The hydrated polymer encircling the lipid bilayer is modelled as a concentric cylindrical layer (belt), extending the full height of the tail region of the bilayer but not covering the headgroup layers. As final refinement of the fitting, parameters for scale and bicelle model were held and parameters of the ellipsoid allowed to vary. The size of the polymer aggregates decreased compared to those of the free polymer alone in solution, which is

expected: due to the loss of some of the polymer into the nanodiscs. The amount of free polymer in solution therefore decreased, decreasing the size of the structures.

During fitting as many parameters as possible were pre-calculated and held, to reduce the number of free parameters in the model. The scattering length density of the SMI polymer, the lipid tails, lipid headgroups and the solvent were all calculated and held during fitting (see Table S1) while the lipid tail thickness and headgroup thickness were set to values previously determined for DMPC bilayers[14]. The background was set using the observed experimental background at high Q. The fitted parameters were therefore the scale factor for the ellipsoid model corresponding to the free polymer, and for the bicelle model, the scale factor, the core radius, the polydispersity in the core radius, the belt thickness and belt region scattering length density were fitted. Errors in fitted quantities were determined by observing the effect of variation of the fitted parameters and correspond to the amount of variation possible before the quality of the fit became worse, determined by an increase in the minimized chi-squared parameter. From the fitting of the belt region scattering length density it is possible to calculate the hydration of the belt as a linear combination of the scattering length density of both polymer and solvent. The hydration of the polymer belt is found to be ~56%.

Size Exclusion Chromatography with Multi Angle Light Scattering (SEC-MALS)

SEC-MALS experiments were performed as previously described[2]. A Superdex 200 increase 10/300 GL size exclusion column attached to an Äkta purification system (GE Healthcare Life Sciences, UK) was equilibrated with 2 column volumes of 50 mM sodium acetate pH 5, 200 mM NaCl. In line absorbance measurements at 280 and 254 nm were used to calibrate the delay volume between the column and the MALS detector. MALS measurements were performed using a Dawn Helios II (Wyatt Technologies, UK) equipped with a 633 nm He-Ne laser with static light scattering detectors positioned at 18 angles radially about the flow cell. The MALS detector at 110 ° has been replaced with a quasi-elastic light scattering (QELS) detector. DMPC SMILPs were prepared as described above and centrifuged at 16,000 × *g* for 10 minutes to remove any contaminant particulate matter. 70 µL of each sample was loaded onto the column which was run with a flow rate of 0.7 mL/min.

The analysis of SEC-MALS data has been discussed in detail elsewhere. We analyzed the data for DMPC SMILPs using a refractive index increment, *dn/dc*, value of 0.16 for saturated phospholipids. As a large proportion of the scattering volume of DMPC SMILPs comprises of

SMI, as determined by SAXS, the masses obtained are estimations for an equivalent particle composed purely of DMPC. Analysis was performed using ASTRA software (Wyatt Technologies, Suffolk, UK) to obtain weight averaged and number averaged molar masses, M_w and M_n, respectively using the Zimm equation[15,16]. The polydispersity index is defined as the ratio of M_w/M_n. Hydrodynamic radii were calculated by measuring the autocorrelation function across the peak and analyzing each curve by cumulant analysis[9] to give a mean Z-average R_h.

Turbidity Measurements

Turbidity measurements were used to measure the solubility of DMPC SMILPs and DMPC SMALPs as a function of the concentration of divalent cations and pH.

For divalent cation stability measurements, DMPC SMILPs and DMPC SMALPs were prepared as described above using 50 mM sodium acetate, 200 mM NaCl, pH 5.0 for SMILPs and 50 mM tris, 200 mM NaCl, pH 8.0 for DMPC SMALPs. Nanodiscs were diluted to 3.22 mg/mL DMPC and 0.5 % (w/v) copolymers in the appropriate buffers. A dilution series was prepared of $MgCl_2$ and $CaCl_2$ from 200 to 2 mM in acetate (for SMILPs) and tris (for SMALPs) buffers as described above. Both the nanodisc solutions and the divalent cation dilution series were centrifuged at 14000 × *g* for 10 minutes to remove any large contaminating matter. 100 µL of diluted nanodiscs were mixed with 100 µL of the divalent cation dilution series in a flat-bottomed 96-well plate and incubated, shaking, at room temperature for 30 mins. The OD was measured at 620 nm for each sample and a background OD subtracted for an identical solution containing no nanodiscs.

To measure the turbidity of DMPC SMILPs and SMALPs as a function of pH, nanodiscs were prepared as described above in 50 mM sodium phosphate, 200 mM NaCl at pH 5.0 for SMILPs and pH 8.0 for SMALPs in order to negate buffer-dependent effects. Nanodiscs were diluted to 0.33 mg/mL DMPC, 0.1 % (w/v) copolymer. 0.2 M HCl or 0.2 M NaCl was added dropwise to SMILPs and SMALPs, respectively, with the solution stirring at room temperature. The pH measured with a probe and was allowed to equilibrate for 5 minutes at each pH point. Three 1 mL aliquots of the solution was transferred to separate 1 cm path length, optically clear poly(styrene) disposable cuvettes and the OD measured in triplicate at 620 nm using a Ultrospec™ 2100 UV-Vis spectrophotometer (GE Healthcare, UK). All turbidity experiments were performed in triplicate.

Preparation of *Escherichia coli* (*E. coli*) BL21(DE3) Membranes

E.coli BL21 (DE3) cultures were inoculated from a single colony on an LB-agar plate and grown in 15 mL LB at 37 °C for 16 hours. A 1 % (v/v) inoculum was used to inoculate 1 L LB. This culture was grown at 37 °C until stationary phase was reached, monitored by measuring absorbance at 600 nm. Cells were isolated by centrifugation at 7000 × *g* for 10 minutes at 4 °C. Pelleted cells were washed in PBS and resuspended in 3.5 mL lysis buffer (50 mM Tris, 5 % (w/v) glycerol, 2 mM EDTA pH 7.5 containing 1 Pearce EDTA-free protease inhibitor tablet per 50 mL) per gram of cells at 4 °C. Cells were lysed by 5 passes through an Emmulsiflex C3 cell disruptor at 4 °C. Cell debris is removed by centrifugation at 11000 × *g* for 30 minutes at 4 °C. Supernatant was removed and membranes isolated by centrifugation at 100000 × *g* for 60 minutes at 4 °C. Supernatant was removed and the wet weight of membranes weighed.

Solubilization of *E. coli* Membranes

E. coli membranes were washed 3 times in phosphate buffered saline (PBS) to remove trace soluble components. Membranes were resuspended to a concentration of 60 mg/mL in either 50 mM sodium acetate, 200 mM NaCl, pH 5.0 or 50 mM sodium phosphate, 200 mM NaCl, pH 7.0. DDM and copolymer stock solutions were made under the same buffering conditions to 2 % (w/v) for DDM and 5 % (w/v) for both SMA and SMI. Membrane resuspensions were mixed with the DDM/ copolymer stocks in a 1:1 ratio and allowed to equilibrate for 2 hours at room temperature. In addition, membranes were diluted to 30 mg/mL with each of the buffers containing no solubilization agent as a control. Insoluble material was removed by ultracentrifugation at 100,000 ×*g* for 45 minutes at 4 ℃. The supernatant, corresponding to the solubilized material, was removed. The insoluble pellets were washed three times in PBS and then resuspended in the same volume of 2 % (w/v) sodium dodecyl sulfate (SDS). The soluble and insoluble fractions were then analyzed by SDS-poly(acrylamide) gel electrophoresis (PAGE) using Criterion XT graduated 4 – 12 % acrylamide bis-tris gels following standard protocols. Gels were stained overnight using InstantBlue protein stain.

Purification of SMALP-solubilized and SMILP-solubilized *E. coli* ZipA

Expression and purification of ZipA protein using SMA 2000 polymer was carried out as described previously[17]. Briefly, ZipA was overexpressed in BL21 E.coli, membranes harvested and solubilized at 30 mg/ml wet weight with 2.5% (w/v) SMA 2000 in buffer A (20mM Tris, 150mM NaCl, pH 8.0). Solubilized protein was harvested by ultracentrifugation (100,000g, 20 min, 4°C), and mixed with Ni-NTA resin (100 µl bed volume per ml solubilized protein) overnight

at 4°C. Resin was washed 5 times with 10 bed volumes of buffer A supplemented with 20mM imidazole, then twice with 10 bed volumes of buffer A supplemented with 40mM imidazole. ZipA was eluted in 6 fractions of 0.5 bed volume using buffer A supplemented with 200mM imidazole. Solubilization and purification using SMI 2000 was the same except that all buffers were at pH 6.5 and the concentration of NaCl was increased to 300mM. Elution fractions were analyzed by SDS-PAGE as described above.

HEK 293T Cell culture and transfection

HEK 293T cells were routinely cultured in Dulbecco's modified Eagles medium (DMEM) containing L-glutamine (2 mM), D-glucose (4500 mg/l) and sodium pyruvate (1 mM) supplemented with 10 % (v/v) fetal calf serum (FCS) in humidified 5 % (v/v) CO_2 in air at 37 °C. For radioligand binding assays, cells were seeded at a density of ~5 x 10^5 cells/100 mm dish and transfected after 48 h. Transfection was essentially as described previously[18]. Briefly, cells were transfected with either human $A_{2A}R$ or human $V_{1a}R$ cDNA in pcDNA3.1(+) using a mixture of 5 µg DNA, 60 µl polyethyleneimine (10 mM) and 1 ml 5 % glucose solution, which was incubated for 30 min at room temperature before addition to an appropriate final volume of full media. GPCR-expressing HEK 293T cells were used 48 h post-transfection.

Radioligand binding assays

Binding assays with $A_{2A}R$ used [^{3}H]ZM241385 (1 nM) as tracer and for the $V_{1a}R$ [^{3}H]AVP (1 nM) was used as tracer. Non-specific binding was defined in a parallel incubation containing a saturating concentration (1 µM) of unlabeled ZM241385 or AVP, respectively. Radioligand alone (for determining total binding), or radioligand plus unlabeled competing ligand (for determining non-specific binding), was added to the culture medium of HEK 293T cells transiently expressing either $A_{2A}R$ or $V_{1a}R$ 48 h post-transfection and incubated at 37 °C . After incubation for 30 min for $A_{2A}R$-expressing membranes or 90 min for $V_{1a}R$-expressing membranes to establish equilibrium, the medium was removed and cells washed three times with ice-cold PBS. Receptors were SMI-solubilized by addition of 1 ml of 20 mM HEPES, 1 mM EGTA, 1 mM magnesium acetate pH 7.4 with 5 % (w/v) SMI, 5 units/ml of benzonase supplemented by the Roche cOmplete (EDTA-free) protease inhibitor cocktail and incubated for 1 h at 37 °C before centrifugation at 100,000 x g for 1 h. The extracted $A_{2A}R$-SMILP and $V_{1a}R$-SMILP were present in the supernatant. Bound ligand in samples of supernatant was quantified by liquid scintillation counting using a

PerkinElmer Tri-Carb 2810 TR liquid scintillation analyzer with HiSafe3 (Perkin Elmer, Wokingham) as cocktail. Specific binding to the SMI-solubilized receptors was calculated by subtracting non-specific binding from total binding.

SUPPORTING DATA

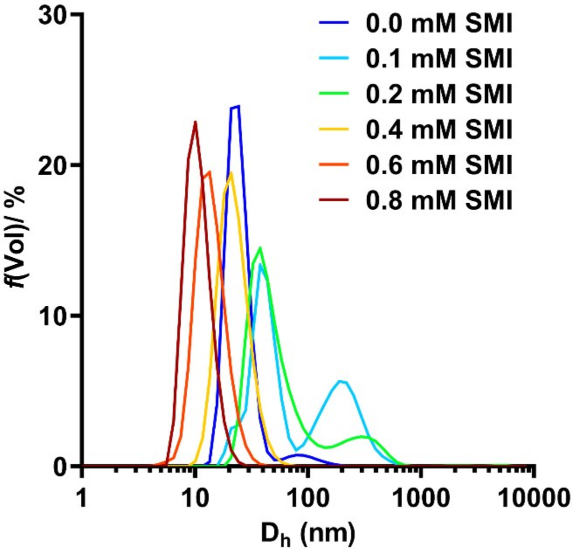

Figure S1: Volume weighted particle size distribution data measured using DLS at SMI concentrations below the polymer concentration required to initiate solubilization of 7.5 mM DMPC, c_S^{SAT}, the saturation boundary. Before the addition of SMI, DMPC is present as small unilamellar vesicles. Upon addition of low concentrations of SMI below c_S^{SAT} (0.1 mM SMI), SMI induces aggregation of DMPC. As c_S^{SAT} is surpassed, the distribution shifts towards smaller diameters. As c_S^{SOL} is approached, a sharp peak of a smaller hydrodynamic diameter (D_h) appears as the proportion of DMPC present as SMILPs increases.

Model	[DMPC] / mg/mL	7	5	3
	**Molar Ratio SMI:DMPC**	**0.54**	**0.75**	**1.26**
**Poly-core bicelle**	**Scale**	$(2.9 \pm 0.5) \times 10^{-12}$	$(2.5 \pm 0.5) \times 10^{-12}$	$(5.0 \pm 0.5) \times 10^{-12}$
	**Core Radius/ Å**	12 ± 2	10 ± 2	7 ± 2
	**Polydispersity Index**	0.18 ± 0.02	0.18 ± 0.02	0.18 ± 0.02
	**Core Length/ Å**	27.56 *		
	**SMI belt Thickness/ Å**	16 ± 2	18 ± 2	17 ± 2
	**Headgroup Thickness/ Å**	8.00 *		
	**Scattering Length Density (SLD) Core/ $\times 10^{-6}$ Å^{-2}**	8.00 *		
	**SLD Headgroup/ $\times 10^{-6}$ Å^{-2}**	12.70 *		
	**SLD SMI Belt/ $\times 10^{-6}$ Å^{-2}**	10 ± 1		
	**SLD Solvent/ $\times 10^{-6}$ Å^{-2}**	9.46 *		
	**Incoherent Background/ cm^{-1}**	7.00×10^{-13} *	2.00×10^{-13} *	4.00×10^{-13} *
**Ellipsoid**	**Scale**	$(7.2 \pm 0.5) \times 10^{-13}$	$(6.0 \pm 0.5) \times 10^{-13}$	$(2.1 \pm 0.5) \times 10^{-12}$
	**Radius (a) rotation axis/ Å**	10 ± 2	10 ± 2	5 ± 2
	**Radius (b) axis/ Å**	11 ± 2	11 ± 2	13 ± 2
	**SLD Free SMI/ $\times 10^{-6}$ Å^{-2}**	10.90 *		
	**SLD Solvent/ $\times 10^{-6}$ Å^{-2}**	9.46 *		
	**Incoherent Background/ cm^{-1}**	0.00 *		

Table S1. Fitting parameters used to fit SAXS data of SMILPs to a model of a poly-core bicelle. To account for free polymer in solution, this model was merged with that for an ellipsoid. Parameters marked with * were fixed throughout the fitting procedure.

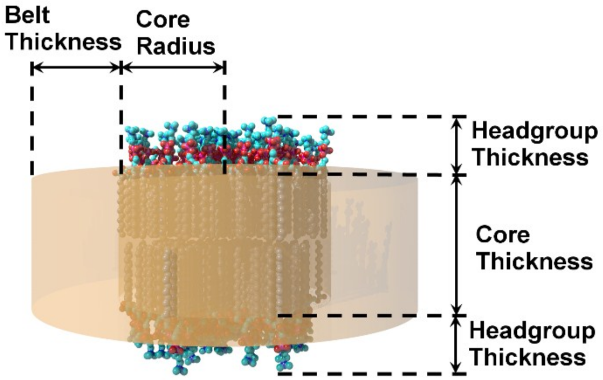

Figure S2. A rendered representation of the poly-core bicelle model used to fit SAXS data with the labelled dimensions corresponding to parameters in Table S1.

**DMPC concentration /mg ml^{-1}**	**0**	**3**	**5**	**7**
**Molar ratio [SMI]:[DMPC]**	**N/A**	**1.26**	**0.75**	**0.54**
**M_W / kDa**	34.2 ± 0.8	91.4 ± 0.7	117.6 ± 0.3	139.8 ± 0.3
**M_n / kDa**	33.5 ± 0.8	90.0 ± 0.7	115.9 ± 0.3	138.2 ± 0.3
**PDI**	1.02 ± 0.03	1.02 ± 0.01	1.02 ± 0.01	1.01 ± 0.01
**Hydrodynamic Diameter / nm**	5.10 ± 0.31	7.24 ± 0.25	8.04 ± 0.23	8.64 ± 0.21

Table S2. Parameters obtained through analysis of SEC-MALS chromatograms of SMI and SMILPs formed at three different SMI:DMPC ratios shown in Figure 3e.

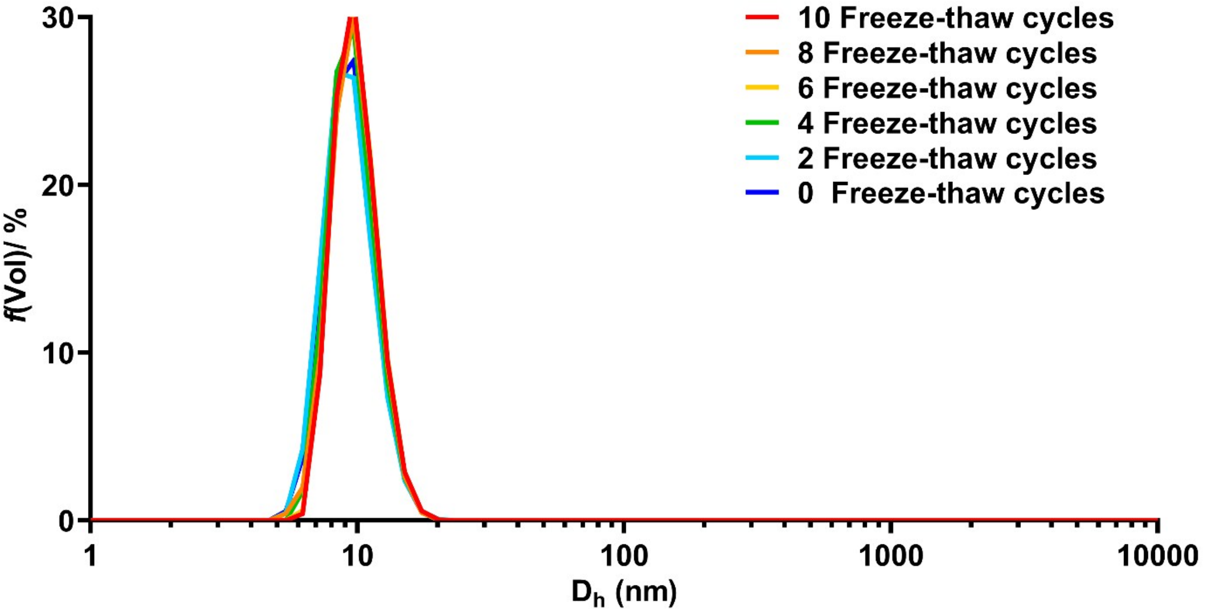

Figure S3. DLS particle size distribution data for SMILP nanodiscs subjected to successive freeze-thaw cycles. After 10 freeze-thaw cycles, only a small shift of the distribution was observed. No large aggregates were observed, suggesting that SMILPs remain intact through multiple freeze-thaw cycles.

SUPPORTING REFERENCES

1 S. C. Lee, T. J. Knowles, V. L. G. Postis, M. Jamshad, R. A. Parslow, Y.-P. Lin, A. Goldman, P. Sridhar, M. Overduin, S. P. Muench and T. R. Dafforn, *Nat. Protoc.*, 2016, **11**, 1149–1162.

2 S. C. L. Hall, C. Tognoloni, G. J. Price, B. Klumperman, K. J. Edler, T. R. Dafforn and T. Arnold, *Biomacromolecules*, , DOI:10.1021/acs.biomac.7b01539.

3 C. Vargas, R. C. Arenas, E. Frotscher and S. Keller, *Nanoscale*, 2015, **7**, 20685–20696.

4 R. Cuevas Arenas, J. Klingler, C. Vargas and S. Keller, *Nanoscale*, 2016, **8**, 15016–15026.

5 A. O. Oluwole, B. Danielczak, A. Meister, J. O. Babalola, C. Vargas and S. Keller, *Angew. Chem. Int. Ed Engl.*, 2017, **56**, 1919–1924.

6 A. Grethen, A. O. Oluwole, B. Danielczak, C. Vargas and S. Keller, *Sci. Rep.*, , DOI:10.1038/s41598-017-11616-z.

7 G. Mie, *Ann. Phys.*, 1908, **330**, 377–445.

8 P. A. Hassan, S. Rana and G. Verma, *Langmuir*, 2015, **31**, 3–12.

9 D. E. Koppel, *J. Chem. Phys.*, 1972, **57**, 4814.

10 M. Basham, J. Filik, M. T. Wharmby, P. C. Y. Chang, B. El Kassaby, M. Gerring, J. Aishima, K. Levik, B. C. A. Pulford, I. Sikharulidze, D. Sneddon, M. Webber, S. S. Dhesi, F. Maccherozzi, O. Svensson, S. Brockhauser, G. Náray and A. W. Ashton, *J. Synchrotron Radiat.*, 2015, **22**, 853–858.

11 L. Feigin and D. Svergun, *Structure Analysis by Small-Angle X-Ray and Neutron Scattering*, Springer, 1987.

12 D. Singh, The Johns Hopkins University, 2008.

13 M. Jamshad, V. Grimard, I. Idini, T. J. Knowles, M. R. Dowle, N. Schofield, P. Sridhar, Y. Lin, R. Finka, M. Wheatley, O. R. T. Thomas, R. E. Palmer, M. Overduin, C. Govaerts, J.-M. Ruysschaert, K. J. Edler and T. R. Dafforn, *Nano Res.*, 2015, **8**, 774–789.

14 N. Kučerka, M. A. Kiselev and P. Balgavý, *Eur. Biophys. J.*, 2004, **33**, 328–334.

15 B. H. Zimm, *J. Chem. Phys.*, 1948, **16**, 1093–1099.

16 P. Wyatt, *Anal. Chim. Acta*, 1993, **272**, 1–40.

17 K. A. Morrison, A. Akram, A. Mathews, Z. A. Khan, J. H. Patel, C. Zhou, D. J. Hardy, C. Moore-Kelly, R. Patel, V. Odiba, T. J. Knowles, M. -u.-H. Javed, N. P. Chmel, T. R. Dafforn and A. J. Rothnie, *Biochem. J.*, 2016, **473**, 4349–4360.

18 M. Jamshad, J. Charlton, Y.-P. Lin, S. J. Routledge, Z. Bawa, T. J. Knowles, M. Overduin, N. Dekker, T. R. Dafforn, R. M. Bill, D. R. Poyner and M. Wheatley, *Biosci. Rep.*, 2015, **35**, e00188.
